# Recent advances in understanding and managing postoperative respiratory problems

**DOI:** 10.12688/f1000research.16687.1

**Published:** 2019-02-18

**Authors:** Matthias Eikermann, Peter Santer, Satya-Krishna Ramachandran, Jaideep Pandit

**Affiliations:** 1 Department of Anesthesia, Critical Care and Pain Medicine, Beth Israel Deaconess Medical Center, Harvard Medical School, Boston, USA; 2 Nuffield Department of Anaesthetics, Oxford University Hospitals NHS Foundation Trust, Oxford, UK

**Keywords:** respiration, hypoxia, hypercapnia, ventilation

## Abstract

Postoperative respiratory complications increase healthcare utilization (e.g. hospital length of stay, unplanned admission to intensive care or high-dependency units, and hospital readmission), mortality, and adverse discharge to a nursing home. Furthermore, they are associated with significant costs. Center-specific treatment guidelines may reduce risks and can be guided by a local champion with multidisciplinary involvement. Patients should be risk-stratified before surgery and offered anesthetic choices (such as regional anesthesia). It is established that laparoscopic surgery improves respiratory outcomes over open surgery but requires tailored anesthesia/ventilation strategies (positive end-expiratory pressure utilization and low inflation pressure). Interventions to optimize treatment include judicious use of intensive care, moderately restrictive fluid therapy, and appropriate neuromuscular blockade with adequate reversal. Patients’ ventilatory drive should be kept within a normal range wherever possible. High-dose opioids should be avoided, while volatile anesthetics appear to be lung protective. Tracheal extubation should occur in the reverse Trendelenburg position, and postoperative continuous positive airway pressure helps prevent airway collapse. In combination, all of these interventions facilitate early mobilization.

## Introduction

Postoperative respiratory complications commonly occur, with an incidence of up to approximately 10% in general surgery ^1– 4^ (even higher with thoracic surgery ^5^). Complications include post-extubation hypoxemia, reintubation, acute respiratory failure, pulmonary edema, pneumonia, and atelectasis. These increase hospital length of stay, unplanned ICU admissions, hospital readmissions, mortality, and costs ^6– 11^. For example, respiratory failure after abdominal surgery can increase 30-day mortality 10-fold ^6^.

## Pathophysiology

Pathologically, we can characterize respiratory complications as being due to respiratory muscle dysfunction or as a primary airway disease. The latter can in turn be subdivided into upper airway-related complications, such as reintubation of an obstructive sleep apnea (OSA) patient, or pulmonary complications, such as pulmonary edema.

**Figure 1.**
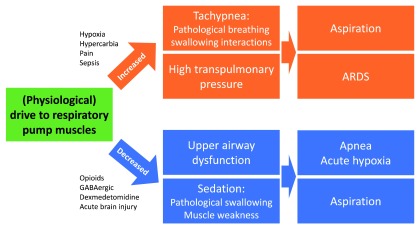
Effects of respiratory drive on perioperative respiratory complication risk. Changes in respiratory drive play a key role in the development of postoperative respiratory complications. Both increases and decreases in respiratory drive are potentially harmful and can affect the risk of aspiration. In addition, an increase in respiratory drive, for example during hypercapnic respiratory failure, can lead to high transpulmonary pressure during inspiration, which increases lung stress. Sedation commonly leads to upper airway dysfunction, resulting in insufficient respiration (hypopnea/apnea) but also affects the breathing–swallowing coordination and pharyngeal muscle strength, both of which contribute to pharyngeal dysfunction and increased risk of aspiration ^12^. Supplementation of inhaled carbon dioxide was shown to reverse upper airway collapsibility induced by propofol ^13^, but excessive hypercapnia increases the likelihood of pathological swallowing ^14^. Thus, perioperative physicians need to balance their interventions to keep ventilator drive within normal limits. ARDS, acute respiratory distress syndrome.

Both respiratory muscle dysfunction and airway disease can develop as a consequence of an imbalance in ventilatory drive. Both increases and decreases in ventilatory drive are potentially harmful and may, for example, increase the risk of aspiration by negatively affecting the interaction between breathing and swallowing ( Figure 1). Sedation due to opioid and anxiolytic therapy commonly leads to upper airway dysfunction, resulting in insufficient respiration (hypopnea/apnea), but also affects the breathing–swallowing coordination and pharyngeal muscle strength, both of which contribute to pharyngeal dysfunction and increased risk of aspiration ^12^. In turn, an increase in respiratory drive (e.g. during hypercapnic respiratory failure) can lead to high transpulmonary pressure during inspiration, which increases lung stress. Supplementation of inhaled carbon dioxide was shown to reverse upper airway collapsibility induced by propofol ^13^, but excessive hypercapnia increases the likelihood of pathological swallowing ^14^. Thus, perioperative physicians need to balance their interventions to keep ventilatory drive within normal limits. Upper airway collapse can lead to desaturation, atelectasis, and respiratory failure. Patency of the upper airway depends on competing dilating versus collapsing forces ^15, 16^. The former includes the pharyngeal dilator muscles (genioglossus and tensor palatini) and caudal traction on the airway from lung expansion (which can be improved by positive end-expiratory pressure [PEEP]). Sedatives, opioids, or even delirium can decrease airway dilator muscle tone. Dilating forces are influenced by atelectasis or the inevitable supine position of surgery. In contrast, collapsing forces include external pressure from surrounding soft tissue, which is increased in the presence of edema, obesity, blood clots, and tumors or in the supine position.

Remarkably, perhaps, significant postoperative pulmonary edema is reported in up to 1–2% of patients ^9^, and causes include negative pressure pulmonary edema, fluid shifts, and, rarely, neurogenic edema in acute hypertension or after cerebral injury ^17^.

More common than edema is atelectasis, and its pathophysiology starts minutes after induction ^18^. A reduced regional transpulmonary pressure in dependent lung areas is accentuated by inflammation induced by surgery, bacterial translocation, chest wall restriction, and cephalad diaphragm displacement by surgical retraction. This extends postoperatively, such that a restrictive pattern worsens respiratory mechanics and gas exchange. Pain, high inflation driving pressures, and inflammation all contribute.

Ventilator-induced lung injury has multiple causes. In addition to barotrauma, reduced lung compliance in unrecruited areas causes overinflation of aerated lung tissue in nondependent areas with subsequent “volutrauma”. Cyclical effects lead to “atelectotrauma”. As mentioned above, the release of local proinflammatory mediators also contributes to lung injury “biotrauma” ^19, 20^.

## Recommendations for patient management

Modifiable perioperative factors in patient management are shown in Table 1. All the aforementioned pathophysiological processes make the optimization of ventilation as a protective strategy logical. What is really important, though, is preoperative screening and patient selection. The Score for Prediction of Postoperative Respiratory Complications (SPORC) is useful in this regard, as it relates the probability of re-intubation to ASA score, emergency surgery, heart failure, and pulmonary disease ^21^. However, SPORC does not include factors such as smoking. Smoking is associated with increased risk of postoperative respiratory complications, and smoking cessation before surgery has been shown to decrease adverse respiratory events ^22, 23^.

**Table 1.** Perioperative factors associated with postoperative respiratory complications (PRCs).

Factor	Main findings	Definition of PRC	Cohort	Reference
**Case management**				
**Open vs.** **laparoscopic** **surgery**	Laparoscopy reduced PRCs	Pulmonary infection, ARDS, symptomatic pleural effusion, respiratory insufficiency, pulmonary embolism	1,214 patients undergoing major hepatectomy	Fuks *et al.* ^30^
**General vs.** **regional** **anesthesia**	Neuraxial anesthesia reduced mortality and PRCs	Pulmonary embolism, pneumonia, respiratory depression	9,559 patients undergoing surgery with or without epidural or spinal anesthesia (systematic review)	Rodgers *et al.* ^31^
**Ventilation**				
**Protective** **ventilation**	Intraoperative protective ventilation was associated with lower risk of PRCs	Respiratory failure, reintubation, pulmonary edema, pneumonia	69,265 non-cardiac surgical patients undergoing general anesthesia with endotracheal intubation	Ladha *et al.* ^28^
**Case-** **specific** **PEEP**	Reduced risk of PRCs and hospital length of stay with PEEP ≥5 cm H _2_O in abdominal surgical, but not craniotomy, patients	Respiratory failure, reintubation, pulmonary edema, pneumonia	5,915 major abdominal surgical patients and 5,063 craniotomy patients	de Jong *et al.* ^29^
**FiO _2_**	High intraoperative FiO _2_ was dose-dependently associated with PRCs and mortality	Respiratory failure, reintubation, pulmonary edema, pneumonia	73,922 mechanically ventilated non-cardiac surgical patients	Staehr-Rye *et al.* ^32^
**Pharmacological factors**				
**Volatile** **anesthetics**	Higher doses of inhalational anesthetics were associated with lower risk of PRCs, reduced mortality, and reduced costs	Respiratory failure, reintubation, pulmonary edema, pneumonia	124,497 non-cardiac surgical patients undergoing general anesthesia with endotracheal intubation	Grabitz *et al.* ^33^
**NMBAs**	Postoperative residual block (TOF ratio <0.7) after pancuronium administration was a risk factor for PRCs	Pneumonic infiltrations or atelectasis on chest X-ray	691 patients undergoing abdominal, orthopedic, or gynecological surgery under general anesthesia	Berg *et al.* ^34^
	Intermediate-acting NMBA use was associated with increased risk of PRCs	SpO _2_ <90% with a decrease after extubation of >3%, reintubation	18,579 patients undergoing surgical anesthesia with NMBA use and 18,579 matched reference patients	Grosse-Sundrup *et al.* ^35^
	NMBA use (and neostigmine reversal) was dose-dependently associated with PRCs	Respiratory failure, reintubation, pulmonary edema, pneumonia	48,499 non-cardiac surgical cases with NMBA use	McLean *et al.* ^36^
	NMBA use was associated with increased risk of PRCs	Respiratory failure, pulmonary infection, pulmonary infiltrates, atelectasis, aspiration pneumonitis, bronchospasm, pulmonary edema	22,803 non-cardiac surgical patients undergoing general anesthesia	Kirmeier *et al.* ^37^
**Fluid** **management**	Liberal fluid administration was associated with PRCs	Respiratory failure, reintubation, pulmonary edema, pneumonia (secondary outcome)	92,094 non-cardiac surgical patients undergoing general anesthesia with endotracheal intubation	Shin *et al.* ^38^
	Liberal fluid administration had a higher risk of pneumonia and pulmonary edema; goal-directed therapy had a lower risk of pneumonia	Respiratory failure, pulmonary edema, pneumonia, and pleural effusion (secondary outcome)	5,021 surgical patients enrolled in 35 RCTs (meta- analysis)	Corcoran *et al.* ^39^
**Opioids**	High intraoperative opioid dose was associated with increased readmission rate but not PRCs	Respiratory failure, reintubation, pulmonary edema, pneumonia (secondary outcome)	74,748 surgical patients undergoing general anesthesia	Grabitz *et al.* ^40^
	Most events occurred within 24 hours after surgery and were preventable in most cases	Respiratory depression	357 acute pain claims	Lee *et al.* ^41^
	Opioids and sedatives are independent and additive predictors of the outcome	Cardiopulmonary and respiratory arrest	6,771,882 surgical inpatient discharges	Izrailtyan *et al.* ^42^

ARDS, acute respiratory distress syndrome; FiO _2_, fraction of inspired oxygen; NMBA, neuromuscular blocking agent; PEEP, positive end-expiratory pressure; SpO _2_, peripheral capillary oxygen saturation; RCT, randomized controlled trial; TOF, train of four.

The method of anesthesia induction can be preventative for postoperative complications. Keeping a patient as upright as possible during induction may help optimize mask ventilation and also help during extubation. This approach may prevent atelectasis, which may be especially important in patients with OSA ^24, 25^.

After intubation, lung-protective mechanical ventilation aims to maintain lung recruitment by keeping transpulmonary pressures within the optimal (linear) part of the local pressure–volume curve. Results from ICU patients suggest reduced morbidity and mortality in the setting of acute lung injury ^26, 27^. Typically, a PEEP of at least 5 cm H _2_O and a median plateau pressure of 16 cm H _2_O appear to be the most beneficial ^28^. However, protective effects of PEEP may be very procedure specific, as a PEEP of approximately 5 cm H _2_O in major abdominal surgery is beneficial, whereas this is not matched by effects of the same level of PEEP in neurosurgery ^29^. Also, PEEP must be patient specific: those with poor chest wall compliance need higher levels of PEEP ^43^. Although high FiO _2_ is used to maintain oxygenation, it may also worsen pulmonary function, probably by promoting atelectasis ^32^.

Interestingly, it has been found that an increased average minimum alveolar concentration of volatile anesthetics, including nitrous oxide, improves 30-day mortality and the risk of pulmonary complications ^33^. The adverse influence of neuromuscular blocking agents (NMBAs) is now well established, especially when associated with inadequate reversal ^34– 37, 44, 45^. Monitoring of NMBAs along with reversal guided by neuromuscular transmission is now mandatory according to minimum monitoring guidelines in the UK ^46^. The choice of reversal agent remains controversial; while sugammadex was shown to reduce the incidence of postoperative residual paralysis compared with neostigmine in one randomized controlled trial ^47^, a recent multicenter observational study (POPULAR trial) found no association between the reversal agent used and postoperative respiratory complications ^37^.

With regard to fluid administration, it is the most-restrictive and the most-liberal strategies that have been associated with respiratory complications, whereas moderate regimens appear to be optimal ^38, 39, 48^. Pain is an adverse factor for respiratory complications, but very high doses of opioids are also potentially harmful ^40^. Neuraxial blockade may reduce postoperative morbidity and mortality in subpopulations ^31, 49^, and laparoscopic surgery, which may contribute to better analgesia, further appears beneficial ^30^. Good pain relief also promotes early mobilization, which shortens patients’ length of stay ^50^. Monitoring is important in the detection of early signs of respiratory complications and the decision to admit and observe a patient in the ICU as opposed to the PACU ^51^.

## Conclusions

**Figure 2.**
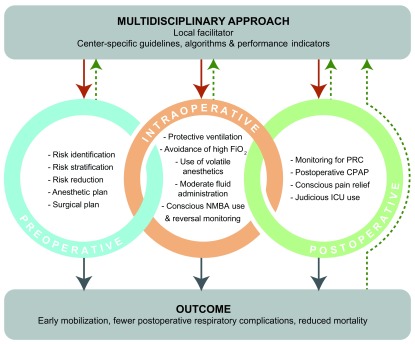
Integration of multilevel guidelines for the prevention of postoperative respiratory complications (PRCs). In a multidisciplinary approach, center-specific guidelines, algorithms, and performance indicators should be developed. Their implementation (red solid arrows) can be facilitated by a local “champion”. Factors concerning the preoperative, intraoperative, and postoperative period need to be addressed, as each can have an impact on outcomes. Periodic review and assessment of processes and outcomes (green dotted arrows) will ensure continuous improvement. CPAP, continuous positive airway pressure; FiO2, fraction of inspired oxygen; ICU, intensive care unit; NMBA, neuromuscular blocking agent.

There is a considerable literature base supporting the individual results highlighted above. What is emerging is the need for the development and implementation of center-specific guidelines, based on algorithms, coupled with key performance indicators developed by multidisciplinary teams ( Figure 2). This can form the basis of a continuous quality improvement program. An important driver in achieving this goal is a local champion or “facilitator”, who can lead the integration of the needed processes.
